# Supplementary material for: Human Cytomegalovirus Seropositivity and Viral DNA in Breast Tumors Are Associated with Poor Patient Prognosis
Source: Cancers (Basel). 2022 Feb 23;14(5):1148. doi: 10.3390/cancers14051148 (PMC8909033; doi:10.3390/cancers14051148)
Supplement: Supplementary file 1 [file cancers-14-01148-s001.zip › cancers-1601292-Supplementary.pdf]

cancers MDPI

Supplementary Material: Human Cytomegalovirus Seropositivity and Viral DNA in Breast Tumors are Associated with Poor Patient Prognosis

Zelei Yang, Xiaoyun Tang, Maria Eloisa Hasing, Xiaoli Pang, Sunita Ghosh, Todd P.W. McMullen, David N. Brindley* and Denise G. Hemmings*

Table S1. Primer sequences for nested PCR.

Primers	Thermocycling conditions (30 cycles)	Product size (bp)	Primer sequence
HCMV *IE1* external	94°C-45 sec<br>55°C-45 sec<br>72°C-45 sec	373	Forward:<br>5′-GGTCACTAGTGACGCTTGTATGATGACCATGTACGGA-3′<br>Reverse:<br>5′-GATAGTCGCGGGTACAGGGGACTCT-3′
HCMV *IE1* internal	94°C-45 sec<br>50°C-45 sec<br>72°C-45 sec	293	Forward:<br>5′-AAGTGAGTTCTGTCGGGTGCT-3′<br>Reverse:<br>5′-GTGACACCAGAGAATCAGAGGA-3′
HCMV *gB* external	94°C-30 sec<br>58°C-30 sec<br>72°C-60 sec	149	Forward:<br>5′-GAGGACAACGAAATCCTGTTGGGCA-3′<br>Reverse:<br>5′-GTCGACGGTGGAGATACTGCTGAGG-3′
HCMV *gB* internal	94°C-30 sec<br>52°C-30 sec<br>72°C-60 sec	96	Forward:<br>5′-ACCACCGCACTGAGGAATGTCA-3′<br>Reverse:<br>5′-TCTTGCGTTTGAAAGAGGTA-3′

bp: base pairs; *gB*: glycoprotein B; *IE*: immediate early.

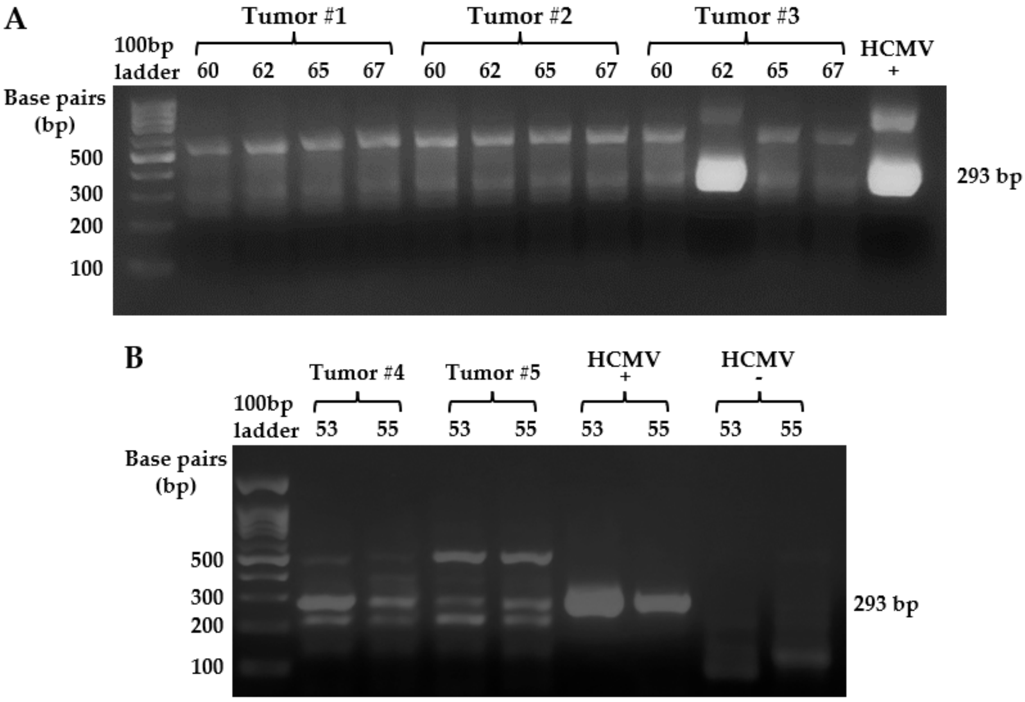

Figure S1. HCMV *immediate early 1 (IE1)* DNA detection using nested PCR with different annealing temperatures. (**A**) The first round of nested PCR using external primers were performed with annealing temperatures including 60, 62, 65 or 67 °C. All second round of PCR reaction with internal primers were performed at annealing temperature of 50 °C.; (**B**) The first round of nested PCR using external primers were performed at annealing temperature of 62 °C. The second round of PCR reaction with internal primers were performed at annealing temperatures of 53 or 55°C. Five human breast tumor samples were illustrated. The PCR products were visualized on 1.5% agarose gel stained with ethidium bromide, with the expected band size of 293 base pair (bp) for a positive detection of HCMV *IE1* DNA. Examples from five human breast tumor samples were illustrated.

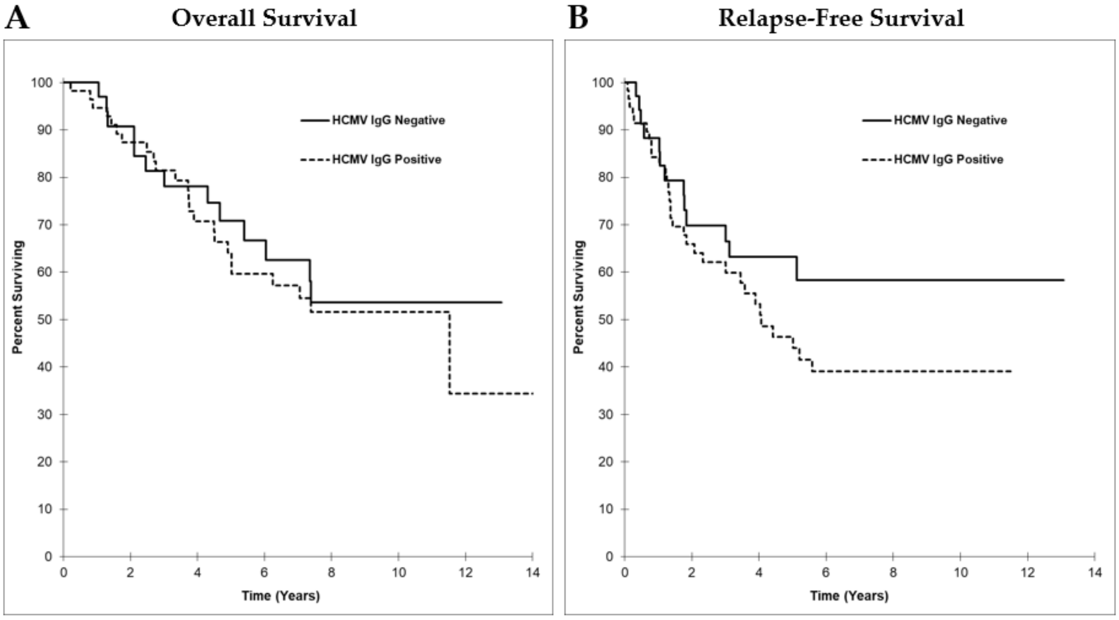

Figure S2. Kaplan–Meier survival curve based on HCMV IgG seropositivity. (**A**) Overall survival and (**B**) Relapse-free survival. $N = 94$. P-values were calculated by the Chi-square analysis. *P*-value was considered significant if < 0.05. (**A**) $p = 0.642$ and (**B**) $p = 0.182$.

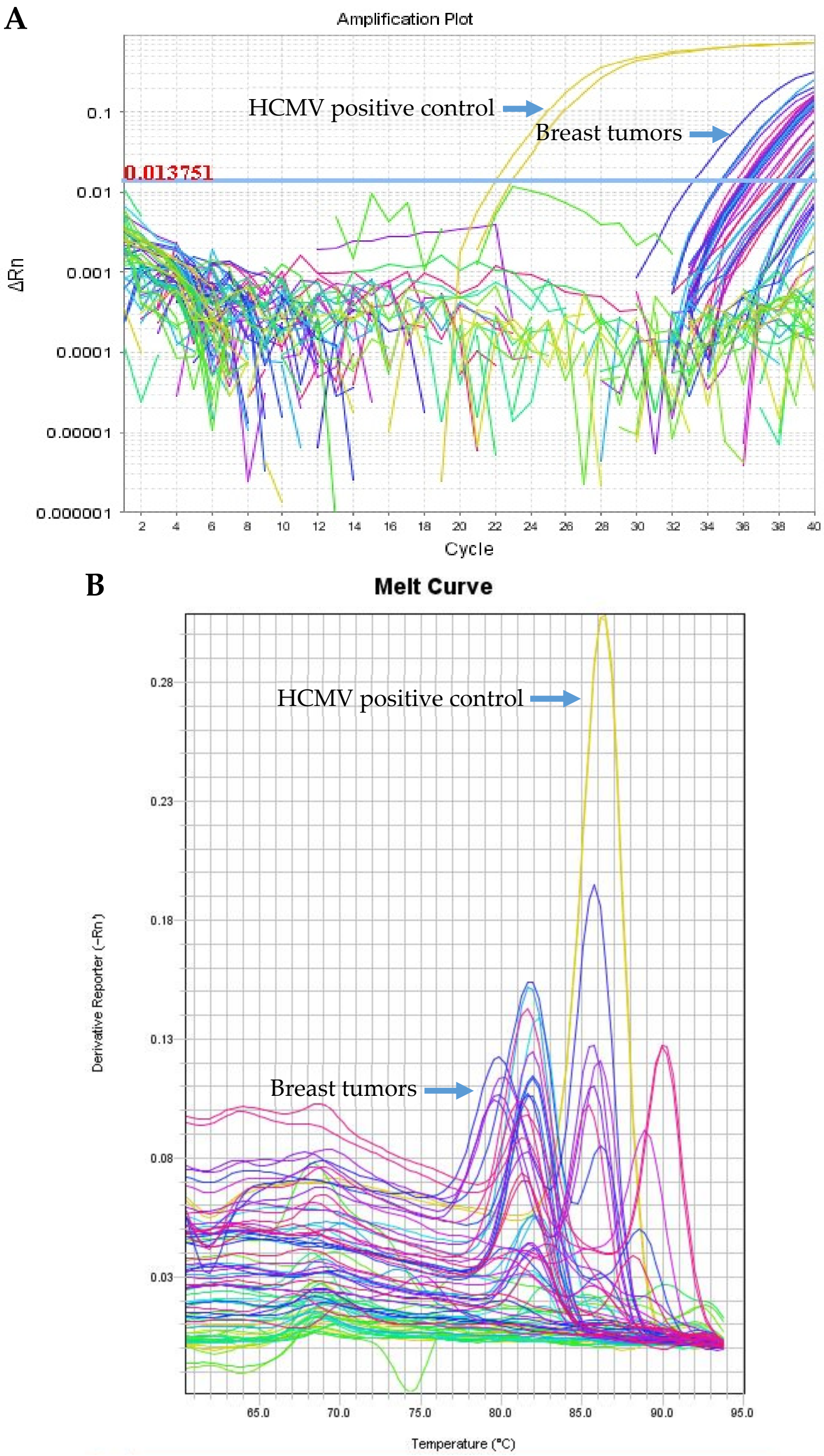

Figure S3. mRNA expression of HCMV *immediate early 1 (IE1)* gene in human breast tumor samples. The expression of IE1 transcript was determined by Quantitative Real-time PCR, with the resulting amplification plot (**A**) and melt curve (**B**) illustrated. The HCMV positive control was depicted in yellow. A total of 136 breast tumors and 10 breast tissues were analyzed, with results from one round of experiment (n = 40) illustrated as example.
